# Sleep Disturbance and Dementia Risk: Findings From 8 Years of Prospective Data

**DOI:** 10.1093/geroni/igaa057.193

**Published:** 2020-12-16

**Authors:** Roger Wong

**Affiliations:** State University of New York Upstate Medical University, St. Louis, Missouri, United States

## Abstract

Recent evidence indicates sleep disturbances increase dementia risk. Despite extensive support for this finding, numerous studies are based on cross-sectional data and no research has examined this relationship using a national sample. The purpose of this study was to analyze how sleep disturbances are associated with dementia risk. This study used eight annual waves (2011-2018) of prospective data from the National Health and Aging Trends Study, a large nationally representative U.S. sample of older adults. At each wave, sleep disturbances were measured as: 1) trouble falling asleep in 30 minutes, 2) trouble falling asleep after waking up early, and 3) taking medication to help sleep. The dependent variable was number of years to a new dementia diagnosis. Multivariate analyses were conducted using the Cox proportional hazards model with survey sampling weights applied for a national sample of 6,800 community-dwelling older adults dementia-free at baseline. After controlling for sociodemographics (age, sex, race, education, etc.) and health (mental health, physical health, chronic disease, etc.), trouble falling asleep in 30 minutes was not associated with dementia risk, (Hazard Ratio [HR]=1.01, p=.98), however, trouble falling asleep after waking up significantly decreased risk (HR=0.40, p<.01), and taking sleep medications significantly increased risk (HR=1.72, p<.01). Our findings suggest mixed evidence on how sleep disturbances are associated with dementia risk, but needing sleep medications may be predictive of elevated dementia risk. Future research should explore pathways or behaviors that may decrease dementia risk among individuals who wake up at night, but have trouble falling back to sleep.

